# Single-Cell Transcriptome Profiling Revealed That Vitrification of Somatic Cloned Porcine Blastocysts Causes Substantial Perturbations in Gene Expression

**DOI:** 10.3389/fgene.2020.00640

**Published:** 2020-07-24

**Authors:** Ling Zhang, Xin Qi, Wei Ning, Luyan Shentu, Tenglong Guo, Xiangdong Zhang, Yunsheng Li, Yangyang Ma, Tong Yu, Jason G. Knott, Zubing Cao, Yunhai Zhang

**Affiliations:** ^1^Anhui Province Key Laboratory of Local Livestock and Poultry, Genetical Resource Conservation and Breeding, College of Animal Science and Technology, Anhui Agricultural University, Hefei, China; ^2^Developmental Epigenetics Laboratory, Department of Animal Science, Michigan State University, East Lansing, MI, United States

**Keywords:** vitrification, pig, somatic cell nuclear transfer, blastocysts, single-cell transcriptome

## Introduction

Cryopreservation of embryos or gametes by slow freezing or vitrification has made great contribution to both the preservation of animal genetic resources and the flexible application of embryo transfer technology. Vitrification has become more and more popular in conservation of mammalian embryos because of its simplicity, speed, low cost, and high success rate. To date, vitrification of embryos in cattle (Taylor-Robinson et al., 2014), mouse (Nakagawa et al., 2018), and human (Wei et al., 2019) is becoming a routine procedure in facilitating the wide application of assisted reproductive technologies in these species. However, vitrification of porcine embryos still remains challenging.

Somatic cloning is valuable in animal genetic resource preservation and human biomedical application. With the increasing demands of genome-edited pigs to model human disease and generate organs for xenotransplantation researches (Niu et al., 2017), there is an urgent need in efficacious production and preservation of porcine somatic cloned embryos. However, incomplete reprogramming of SCNT (Somatic Cell Nuclear Transfer) embryos will reduce the quality of embryos (Zhang et al., 2018), which also reduces the success rate in vitrification of embryos. At present, the survival rate of porcine vitrified early embryos is still very low, and it is necessary to elucidate the mechanisms underlying vitrification of porcine embryos. It would be beneficial to develop useful strategies and optimize vitrification procedure. It was found that excessive lipid droplet content is an important factor leading to high sensitivity to low temperature in porcine embryos (Fu et al., 2009). Initial studies have found that high concentrations of lipid droplets can increase the level of apoptosis in vitrified embryos (Men et al., 2006). Further studies have found that the vitrification process affected the levels of oxidative stress and apoptosis-related genes (Chen Y.N. et al., 2018), and by rescuing these pathways, the survival rate of pig embryos after thawing can be significantly improved. Therefore, it is necessary to explore the effects of vitrification in the expression of key genes important for the development of porcine cloned embryos. To date, the transcription level of genes in porcine oocytes and early embryos by vitrification is mainly examined by real-time quantitative PCR (qRT-PCR). As revealed by using qRT-PCR, vitrification will affect the apoptosis-related genes *TNF-*α, *caspase-8, caspase-9*, and *Bcl-2* (Chen Y.N. et al., 2018); oxidative stress-related genes *SOD1* and *SOD2* (Castillo-Martin et al., 2015); lipolysis-related genes *LIPE, PLPLA* (Gomez et al., 2017) and *IGF2, IGF2R* (Bartolac et al., 2018); and other genes in porcine embryos. However, qRT-PCR can only detect a small number of genes, while traditional high-throughput sequencing requires a large number of cells. Recently, Smart-seq2 technology has been developed to analyze transcripts at a single cell level (Picelli et al., 2014). This technique has been successfully applied to detect the effect of vitrification on the transcriptome in mouse oocytes (Gao et al., 2017; Lee et al., 2019) and bovine oocytes (Wang et al., 2017; Huang et al., 2018). In particular, by using the Smart-seq2 technique, it was found that vitrification mainly affected apoptosis in mouse oocytes (Lee et al., 2019). In addition, Jia et al. used the Smart-seq2 technique to study the effects of vitrification on the porcine oocyte transcriptome (Jia et al., 2019) and found that 37 genes were differentially expressed in vitrified GV oocytes after IVM. Currently, the effect of vitrification on transcriptomic profiles in porcine somatic cloned blastocysts is still lacking.

In all, it is desirable to explore the molecular mechanism underlying the injury of porcine cloned embryos caused by vitrification. Therefore, in this study, we collected re-expanded porcine cloned blastocysts after vitrification-thawing and fresh porcine cloned blastocysts, then performed single-cell transcriptome sequencing. The smart-seq2 technique was employed to investigate the effect of vitrification on the transcriptional level of porcine cloned blastocysts.

## Methods

Reagents and chemicals were purchased from Sigma (Sigma-Aldrich, St. Louis, MO) unless otherwise stated. All experiments were conducted according to the Institutional Animal Care and Use Committee (IACUC) guidelines under current approved protocols at Anhui Agricultural University.

### *In vitro* Maturation

Ovaries were collected from a local slaughterhouse (Fuyang Furun Meat Co. Ltd., Fuyang City) and were transported to the laboratory at 28 ± 2°C within 5 h. Follicles (2–5 mm) were aspirated using a 10-mL syringe with an 18-G needle. Cumulus–oocyte complexes (COCs) invested with more than two layers of granular cells were collected in Dulbecco's phosphate buffered saline containing 0.1% polyvinylpyrrolidone and penicillin–streptomycin (Gibco, 15140122). COCs were then suspended in a porcine IVM medium with 1 nM melatonin, 16.25 μg/L leukemia inhibitory factor (ProSpec, CYT-191), 16 μg/L insulin-like growth factor-1 (ProSpec, CYT-022), and 32 μg/L fibroblast growth factor-2 (ProSpec, CYT-085) and incubated at 38.5°C and 5% CO_2_ for 42–44 h.

### Production of Somatic Cloned Embryos

Duroc pig fibroblasts derived from adult pig ears were used as donors for making cloned embryos. Metaphase-II stage porcine oocytes were suspended in TCM199 containing 7.5 mg/L cytochalasin B and 2% fetal bovine serum (Gibco 10099). An oocyte was held with its polar body at the 5 o'clock position and enucleated using a microneedle (diameter, 20 μm) to remove the first polar body and one-fifth of the surrounding cytoplasm. After enucleation, a donor cell was injected in the perivitelline space. After recovery for 0.5–1 h in porcine zygote medium-3 (PZM-3) with a CF-150B cell fusion instrument (BLS), a single pulse direct current of 1.56 kV/cm for 100 μs was administered to induce cell fusion and activation. Subsequently, the reconstructed embryos were immediately transferred into a chemically assisted activation medium (PZM-3 with 10 mg/L cytochalasin B and 10 mg/L cycloheximide) and incubated in PZM-3 at 38.5°C and 5% CO_2_ for 4 h. Fusion results were subsequently determined.

### Vitrification of Cloned Embryos

The Cryotop method was used for vitrification as previously described by Kuwayama et al. (2005). Briefly, after two washes in a balanced medium for 2 min, the porcine cloned blastocysts were equilibrated in an equilibrium solution for 8–10 min. A group of three embryos were then transferred into a vitrification solution, loaded onto the Cryotop carrier, and immediately submerged into liquid nitrogen. The entire process involving embryo transfer from the vitrification solution to liquid nitrogen was completed in no more than 1 min and was performed at room temperature. The embryos were stored in liquid nitrogen for at least 2 weeks and then warmed at 39°C. The Cryotop carrier with the embryos was quickly immersed into a warming solution for 1 min, transferred into a diluent solution for 5 min, balanced for 3 min in a handling medium, and then transferred into a new handling medium for 3 min. Warmed embryo was cultured in PZM-3 for 6 h. Embryo survival was determined according to the state of blastocyst re-expansion.

### RNA Amplification and Sequencing Library Preparation

The experiments were repeated three times with both the sample groups and each sample containing one blastocyst. The first is the control group, in which the pig cloned blastocysts were developed to the seventh day (SCNT-CNT). The second is the experimental group, of which the pig embryos developed to the seventh day were vitrified and frozen stored in liquid nitrogen for more than 2 weeks, after which the pig embryos were thawed and expanded during 6 h (SCNT-VT). The lysates were deposited at −80°C, and the RNA was extracted within a week. We used the Smart-seq2 method for first-strand cDNA synthesis to establish a sequencing library. The construction and sequencing of transcriptome libraries were performed at LC-BIO Bio-tech Ltd. (Hangzhou, China) as per the protocol recommended by the vendor. Briefly, mRNAs were first segmented in a fragmentation buffer, followed by first-strand cDNA synthesis. After second-strand synthesis, double-stranded cDNAs were end repaired by T4 DNA polymerase, followed by A-tailing with Klenow DNA polymerases [poly(A)-tail at the 3′-end]. AMPure XP beads were used to clean up the libraries, and PCR was performed to obtain the final sequencing library. The library was sequenced using Illumina Hiseq4000, which produced paired-end libraries with 150 bp (PE150) read length.

### Single-Cell Transcriptomic Analyses

For further analyses, the raw sequencing data were filtered to identify and obtain valid or clean data. Each sample contained ~90% of available data, and the data volume was ~7 G (Supplementary Table 1). Gene expression levels were measured in “fragments per kilobase of exon model per million mapped reads” (FPKM). EDGER was used to compare gene expression levels between the two groups, with thresholds at |log2foldchange| ≥ 1 with the adjusted *p* < 0.05. Significant differential genes were selected for the analysis.

Gene ontology (GO) analyses were performed, which involved the clustering of genes depending on their functions, including analyses of their molecular function, location within the cell (cellular component), and participation in biological processes. Kyoto Encyclopedia of Genes and Genomes (KEGG) was used to obtain an overview of metabolic and regulatory pathways.

### Validation of RNA-Seq Results by Single Cell Real-Time Quantitative PCR

qRT-PCR was performed using Single Cell Sequence Specific Amplification Kit (Vazyme, P621-01) according to the manufacturer's instructions. The details related to primers are present in Supplementary Table 2. Briefly, one blastocyst was added to 5 μL of a reaction mixture, comprising of 2.5 μL 2 × reaction mix, 0.5 μL primer assay pool, 1.9 μL nuclease-free water, and 0.1 μL RT/Taq enzyme. The mixture was immediately incubated for 2 min at −80°C, followed by centrifugation at 1,000 × g for 2 min at room temperature. Subsequently, the sample was incubated at 50°C for 60 min and then at 95°C for 3 min, followed by 17 cycles of 95°C for 15 s and 60°C for 15 min to complete the first round of amplification. Quantitative PCR was performed using AceQ qPCR SYBR Green Master Mix (Vazyme, Q111-02) on ABI StepOne Plus (Applied Biosystems, Foster, USA). Data analysis was carried out using the 2^−ΔΔCt^ method. *EF1*α*1* was used as the housekeeping gene.

### Statistical Analyses

Experiments were repeated more than three times, and SPSS version 17.0 was used for data analyses. Differences between the groups were analyzed by *t*-test. Values are expressed as mean ± standard error of mean; *p* < 0.05 indicated statistical significance.

## Results

### Effect of Vitrification on the Survival Rate of Porcine Cloned Blastocysts

**Figure 1:**
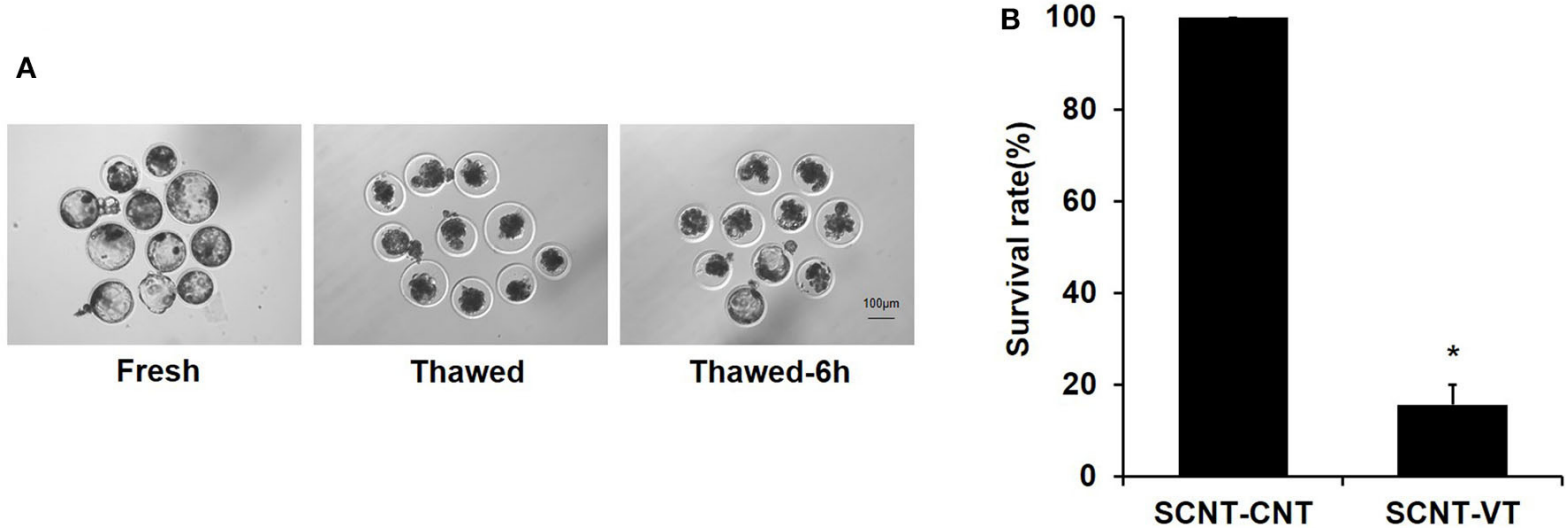
Survival rate of vitrified porcine cloned blastocysts. **(A)** Cloned blastocysts before cryopreservation, 0 h after thawing, and 6 h after thawing. **(B)** Survival rate (%) of control and vitrified/thawed porcine cloned blastocysts (thawing for 6 h). Scale bar = 100 μm. *Indicates significant differences (*p* < 0.05).

Previous studies have shown that the vitrification/thawing process reduces the survival rate of porcine blastocysts (Kikuchi et al., 2016). We noted that the developmental synchronization of cloned embryos was poor. Some embryos were prematurely incubated, and their zona pellucida was damaged, as determined via microscopic observations (Figure 1A). This study was repeated seven times. A total of 72 porcine cloned blastocysts were cryopreserved, of which 12 blastocysts were re-expanded at 6 h after thawing. A significant reduction in the survival rate of cloned blastocysts was noted in response to the vitrification procedure (100% vs. 15.71% ± 4.3%, *p* < 0.05) (Figure 1B). Moreover, porcine cloned blastocysts were very sensitive to damage upon being subjected to the recovery process.

### Identification and Characteristics of Differentially Expressed Genes Between Fresh and Vitrified Porcine Cloned Blastocysts

**Figure 2:**
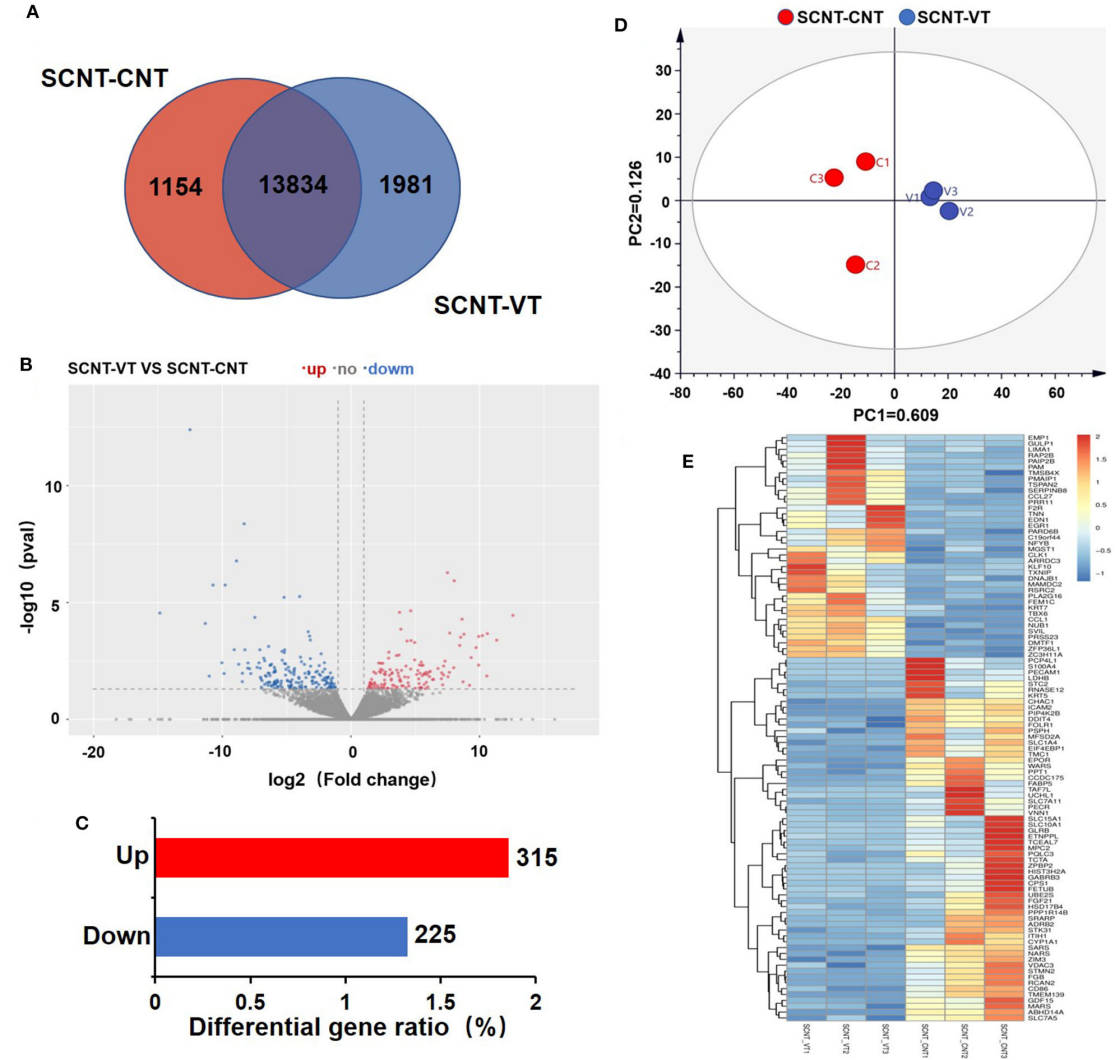
Differentially expressed genes (DEGs) in control and vitrified/thawed porcine cloned blastocysts. **(A)** Venn diagram showing the expression of genes between the control and experimental groups; each circle indicates gene expression between the two groups. **(B)** Volcano plot depicting significant differences in gene expression between the control and experimental groups. **(C)** Bar chart showing DEGs that were up- (red bars) and downregulated (blue bars). **(D)** Two-dimensional principal component analysis showing DEGs in the control and experimental groups. **(E)** Clustering analysis of gene expression levels (measured in “fragments per kilobase of exon model per million mapped reads”). Red indicates upregulation and blue indicates downregulation.

RNA-seq was used to investigate the gene expression, which were measured for each sample in FPKM. All of the six samples showed relatively stable log10(FPKM) values, which were between 1.59 and 2.25 (Supplementary Figure 1A). The density map of sequencing data for each sample was found to be normally distributed and repeatable (Supplementary Figure 1B). Figure 2A depicts a Venn diagram that shows that out of a total of 16,969 genes, 13,834 genes showed significant differences in their expression levels. A total of 1,154 and 1,981 genes were exclusively detected in the SCNT–CNT and SCNT–VT group, respectively.

Between the SCNT–CNT and SCNT–VT groups, we further analyzed 16,969 genes for differential expression, as shown in the Volcano plot in Figure 2B. In this figure, red and blue dots indicate upregulated and downregulated genes, respectively (the adjusted *p* < 0.05). We identified a total of 540 DEGs, of which 315 were upregulated, and 225 were downregulated (Figure 2C). The differential gene expression in the two groups was well-distinguishable by PCA, as shown in Figure 2D (PC1 = 60.9%, PC2 = 12.6%). A heat map was then constructed to visualize the effect of vitrification on gene expression levels (Figure 2E). We randomly selected 100 DEGs. In the heat map, red to blue indicates high-to-low gene expression levels. We found that vitrification significantly upregulated as well as downregulated the expression levels of several genes. As per the RNA-seq results with 315 genes upregulated and 225 downregulated, the |log2| ≥ 2 was used to display the genes that were significantly upregulated (Supplementary Table 3) and downregulated (Supplementary Table 4) as an effect of the vitrification.

**Figure 3:**
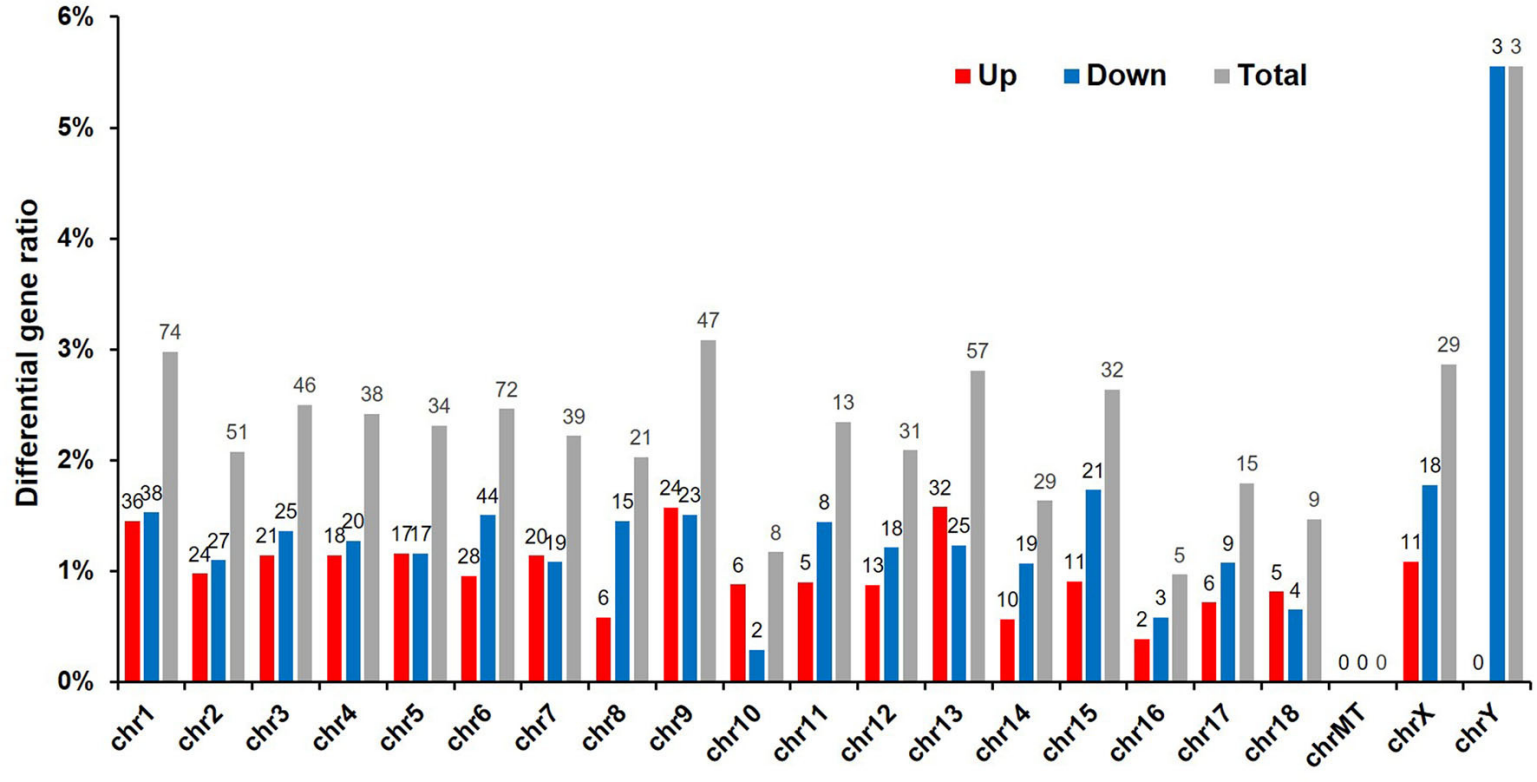
Chromosomal locations of differentially expressed genes (DEGs). Sequencing results led to the identification of 540 DEGs (*p* < 0.05). Red, number of upregulated genes; blue, number of downregulated genes; and gray, total number of genes that were differentially expressed in response to the vitrification/thawing method.

To detect whether vitrification had any effect on porcine chromosomes, we analyzed the location of DEGs in the chromatin. Because the adult boar fibroblasts were used as the donor cell for cloned embryos, and the Y chromosome was detected via the sequencing results, three genes were significantly downregulated and have the highest proportion of affected genes, reaching 6%, indicating that the cloned embryos were successfully constructed. The DEGs caused by vitrification are located on almost all chromosomes, and the number of differential genes on chromosome 1 is the largest (74/540, 15%). The vitrification procedure did not affect the expression level of mitochondrial genes (Figure 3).

### Functional Annotation of Differentially Expressed Genes in Porcine Cloned Blastocysts

**Figure 4:**
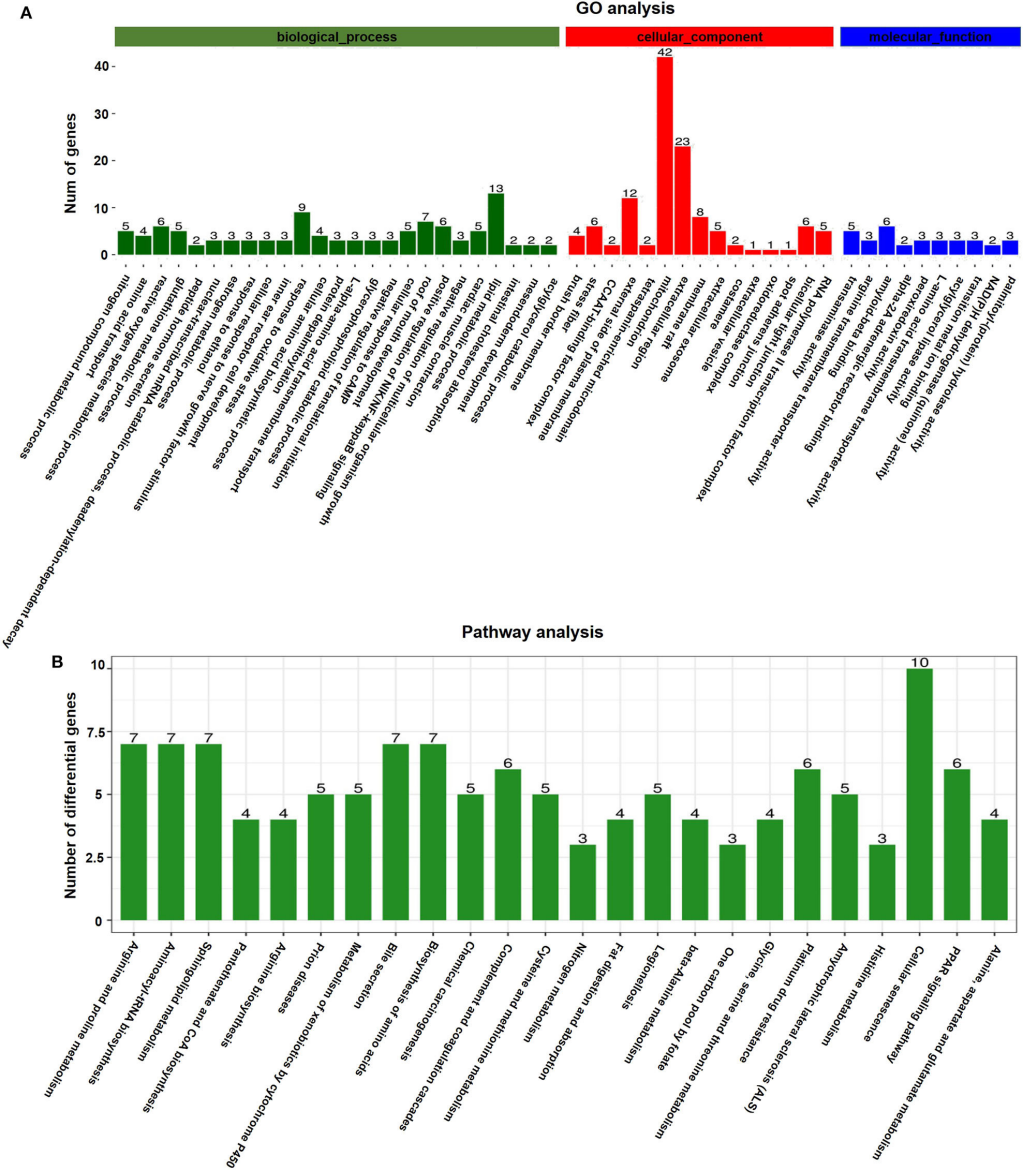
Gene function analyses of vitrified/thawed porcine cloned blastocysts. **(A)** Distribution of gene ontology (GO) categories as per the three major GO domains. Genes were classified as per those related to biological processes (a), cellular components (b), and molecular functions (c). Bars represent the number of sequences in different GO categories. **(B)** KEGG annotation of differentially expressed genes (DEGs); 126 DEGs were subjected to KEGG annotation and were grouped into 24 pathway classes. The vertical axis shows the name of KEGG terms and the horizontal axis displays the number of DEGs. *p* < 0.05 was used as the threshold to select significant KEGG pathways.

GO analyses were applied to the genes that were differentially expressed in response to vitrification. DEGs were classified according to three independent categories: biological process, cellular component, and molecular function. According to the adjusted *p*-value of the selected part term from small to large, the first 15 terms of the biological process and the first 10 terms of the cellular component and molecular function were selected and shown in the article (Figure 4A). In the biological process ontology, the nitrogen compound metabolic process, amino acid transport, ROS metabolic process, and glutathione metabolic process were the most affected by vitrification, while the maximum number of genes related to response to oxidative stress (9 genes) and lipid metabolic process (13 genes) was significantly altered. In the cellular component ontology, the brush border membrane and stress fiber were the most affected by vitrification, while the maximum number of genes related to the mitochondrion (42 genes) and extracellular region (23 genes) was significantly altered. Finally, in the molecular function domain, transaminase activity, arginine transmembrane transporter activity, and amyloid-β binding were the most affected by vitrification. A detailed information pertaining to GO analyses is presented in Supplementary Table 5.

According to KEGG pathway analyses, DEGs were found to be enriched in several key pathways. We identified that a total of 914 genes were differentially expressed in response to the vitrification procedure, which could be mapped to 271 KEGG pathways. Further, 126 DEGs were subjected to KEGG annotation and were grouped into 24 pathway classes (Figure 4B). The arginine and proline metabolism (ko00330, 35 genes), aminoacyl-tRNA biosynthesis (ko00970, 40 genes), and sphingolipid metabolism (ko00600, 42 genes) pathways were the most significantly impacted, and the largest number of genes related to the cellular senescence pathway (ko04218, 124 genes) (Supplementary Figure 2) was affected by the vitrification procedure. Detailed information is presented in Supplementary Table 6.

### Validation of RNA-Seq Results by Single Cell Real-Time Quantitative PCR

**Figure 5:**
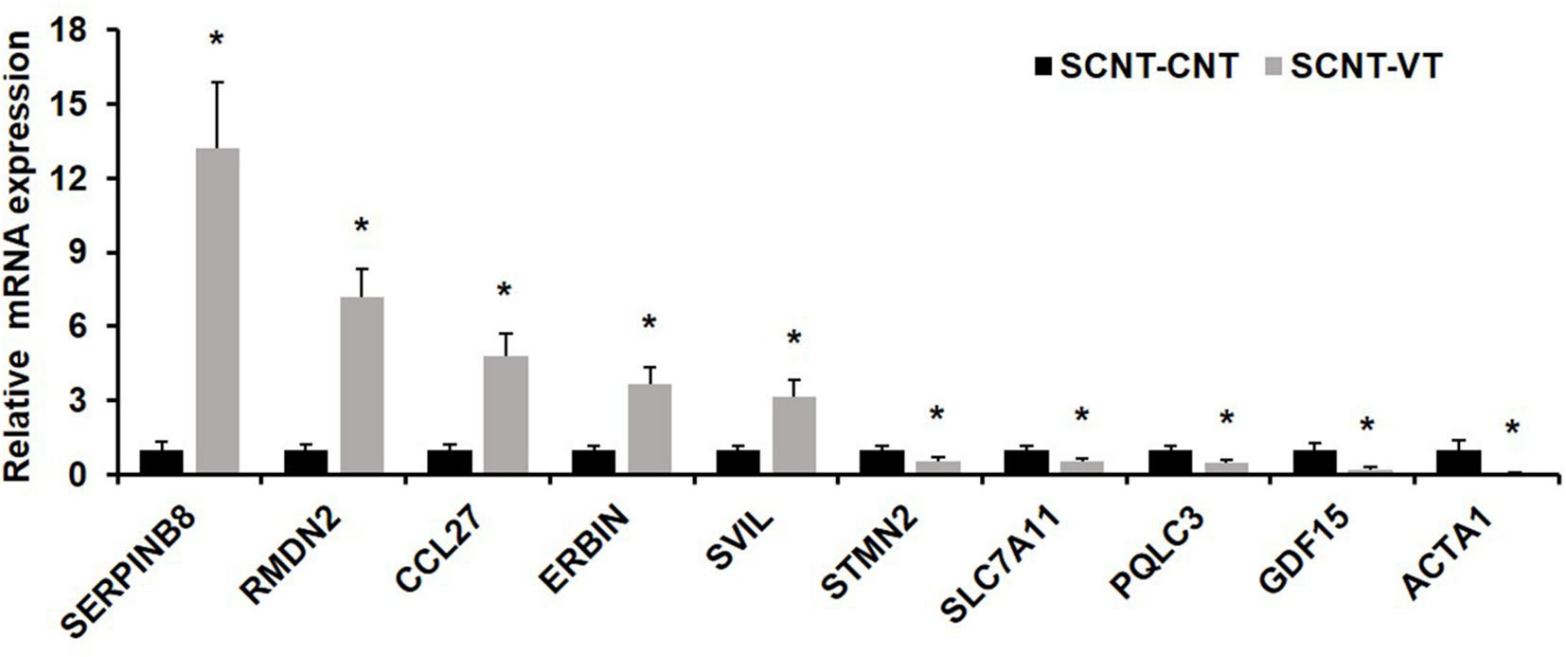
Quantitative real-time PCR (qRT-PCR) of randomly selected differentially expressed genes (DEGs). Ten randomly selected DEGs were chosen and expression levels were validated using qRT-PCR. In response to the vitrification procedure, five genes were significantly upregulated (*SERPINB8, RMDN2, CCL27, ERBIN, SVIL*) and five were significantly downregulated (*STMN2, SLC7A11, PQLC3, GDF15, ACTA1*). *EF1*α*1* was used as the housekeeping gene. Values represent mean ± standard error of mean, and *indicates significant differences (*p* < 0.05).

To verify the results of single-cell sequencing, we randomly selected five upregulated and five downregulated genes from the pool of DEGs, and used a single-cell qRT-PCR quantitative method to verify our results. qRT-PCR showed that the expression pattern of these randomly selected genes was consistent with that determined via RNA-seq (Figure 5).

## Discussion

Vitrification reduces the developmental efficiency of mammalian embryos and even leads to embryo death, apoptosis, and abnormal alterations in mRNA levels. The anomalous changes in gene expression levels in porcine blastocysts may affect subsequent embryo development and implantation. In this study, we report the effects of vitrification on the transcriptomic changes in porcine cloned blastocysts, and we verified our sequencing results using single-cell qRT-PCR. The expression trends were the same in both sets of data. Our sequencing results should contribute toward understanding the mechanisms by which vitrification damages porcine cloned embryos, which in turn should help improve the efficiency of the technique. Cryopreservation efficiency for porcine cloned blastocysts is still evidently very low, though many efforts have been made. The survival rate of frozen-thawed cloned embryos is significantly lower than that for *in vitro* fertilized and parthenogenetic embryos (Gil et al., 2010). The low developmental competence of frozen-thawed cloned embryos could be due to the insufficient reprogramming of somatic cells. In this study, we used adult pig fibroblasts as donor cells. Hua et al. proved that adult pig fibroblasts, as donors, reduce reprogramming efficiency, embryo quality, and developmental capacity compared to young pig fibroblasts (Hua et al., 2016). A high lipid content in porcine embryos can cause higher sensitivity to low temperatures. It has been reported that porcine embryos produced from delipidated oocytes by parthenogenetic activation or handmade cloning (HMC) could be effectively cryopreserved by ultrarapid vitrification (Du et al., 2007). Tecirlioglu et al. proposed HMC as an alternative technique for the generation of cloned offspring in the bovine, considering that the birth and continued survival of clones produced with HMC were equivalent to those produced with conventional nuclear transfer (Tecirlioglu et al., 2005). Another study, which used pig as a model, found that faster embryo development underlies higher blastocyst cryotolerance, providing evidence that blastocoel cavity expansion before vitrification is a robust index of *in vitro*-produced embryo quality and developmental potential (Morato et al., 2016).

The deleterious effects of vitrification on embryos or oocyte cell structure are an important cause of impaired embryo development after thawing (Somfai et al., 2012). Damage to mitochondria tends to increase ROS levels (Herranz and Gil, 2016), resulting in the activation of the apoptosis pathway (Golovach et al., 2017). Vitrification could negatively affect mitochondria in embryos. Our sequencing results revealed that mitochondrial-related genes were significantly affected by vitrification/thawing. The genes encoding *BAD* and *PMAIP1* are important members of the apoptosis pathway. Chen et al. reported that mitochondrial damage is an important factor in the apoptosis of parthenogenetic pig blastocysts (Chen Y.N. et al., 2018). The cell membrane acts as a physical and chemical barrier between the cell and the environment. Vitrification of cloned embryos could markedly affect cell membrane-related genes. For example, this research found that the 20 solute carrier (SLC) family genes were significantly affected by cryogenic preservation. SLC family members are known to transport molecules across the cell. Moreover, it has been reported that *SLC2A3* plays an important role in the ability of bovine blastocysts to withstand cryopreservation, which is often used as an indicator of embryo quality (Kuzmany et al., 2011). Further, via GO analyses, we noted that vitrification not only significantly affected genes related to cellular membrane structure but also those associated with transmembrane transport, the external side of the plasma membrane, and transmembrane transporter activity. Also, the cytoskeleton-related genes were impacted. Collectively, such deleterious effects can lead to the inhibition of embryo development or even miscarriage. Recovery from vitrification often accompanies strong physical damage to subcellular and cellular structures. Our results should contribute toward revealing the mechanism of damage from a genetic perspective in order to help enhance the efficiency of the method.

The vitrification of porcine blastocysts would reduce the survival rate. Moreover, thawing of embryos is associated with certain problems in the development and implantation stages. KEGG pathway analysis revealed significant changes in genes associated with metabolic pathways, such as arginine and proline metabolism; cysteine and methionine metabolism; β-alanine metabolism; glycine, serine, and threonine metabolism; histidine metabolism; alanine, aspartate, and glutamate metabolism; nitrogen metabolism; and sphingolipid metabolism. Such pathways could lead to significant effects on amino acid, protein, and lipid metabolism-related pathways. Dufort et al. revealed alterations in gene expression levels induced by the Cryotop technique in bovine blastocyst-stage embryos, and their results showed that the expression levels of metabolism-related genes were altered in response to cryopreservation (De Oliveira Leme et al., 2016). Amino acid metabolism plays a pivotal role in the embryonic development process. Chen et al. (2018) found that glutamine supplementation enhanced the developmental efficiency of *in vitro*-produced porcine embryos and increased the rate of birth after implantation. Moreover, glycine supplementation has been reported to improve the rate of maturation of porcine oocytes and also their quality post-maturation. This is mainly because glycine metabolism reportedly increases the content of glutathione in the sows of pigs, which improves the antioxidant properties of oocytes. Furthermore, glycine metabolism can induce the expression of *FGFR2* and *HSF1* (Li et al., 2018). Although very high lipid levels can reduce embryo freezing efficiency, lipids are an important source of energy for embryos, playing a key role in embryonic development (Amstislavsky et al., 2019). It has been reported that abnormal metabolism of lipids can cause cell apoptosis and necrosis (Qi et al., 2019). Dos Santos et al. (2019) found that saffron can enhance the *in vitro* development of bovine embryos by increasing lipid metabolism levels. Mitochondria are important metabolic sites, and they affect embryo development via amino acid metabolism, glycolysis, and fatty acid metabolism (Kim and Seli, 2019). Cryopreservation can cause mitochondrial metabolic disorders that increase ROS levels in embryos, adversely affecting developmental competency after thawing (Sefid et al., 2017). Metabolic disorders caused by vitrification may be another cause of disruption of embryo development.

## Conclusions

To summarize, vitrification reduced the survival rate of porcine cloned blastocysts and caused the altered expression of 540 genes. The RNA-seq results provided insights into the mechanisms responsible for damaging porcine cloned embryos at low temperatures. Our findings should help toward improving the cryopreservation efficiency of porcine embryos.

## Data Availability Statement

The datasets generated for this study can be found in the [SRA] [https://www.ncbi.nlm.nih.gov/bioproject/PRJNA634966].

## Ethics Statement

The animal study was reviewed and approved by Institutional Animal Care and Use Committee (IACUC).

## Author Contributions

LZ, XQ, and WN performed experiments. YZ and ZC designed and supervised the project. LS, TG, and XZ provided the materials for experiments. LZ and XQ wrote and edited the manuscript. YL, YM, TY, and JK commented on article writing and revision. All authors have read and agreed to the published version of the manuscript.

## Conflict of Interest

The authors declare that the research was conducted in the absence of any commercial or financial relationships that could be construed as a potential conflict of interest.

## Supplementary Material

Supplementary Figure 1 RNA-Seq data quality. The boxplot **(A)** shows the gene expression level [log10(FPKM)] for each sample. **(B)** Density maps for different samples.

Supplementary Figure 2 Differentially expressed genes in vitrified/thawed porcine cloned blastocysts (SCNTVT) that were involved in the cellular senescence pathway. DAVID analysis showed that the cellular senescence pathway was significantly influenced (p = 0.037). Red indicates genes upregulated in the SCNTVT group: HIPK3, MAP2K3, GADD45B, and ZFP36L1. Blue indicates genes downregulated in the SCNTVT group: EIF4EBP1, CALML5, VDAC3, and SLC25A4.

Supplementary Table 1 Summary of sequencing results in vitrified and fresh porcine cloned blastocyst.

Supplementary Table 2 Porcine-specific primer sequences used in the study.

Supplementary Table 3 Genes upregulated in vitrified porcine cloned blastocyst.

Supplementary Table 4 Genes downregulated in vitrified porcine cloned blastocyst.

Supplementary Table 5 GO analysis of differentially expressed genes in vitrified and fresh porcine cloned blastocyst.

Supplementary Table 6 KEGG analysis of differentially expressed genes in vitrified and fresh porcine cloned blastocyst.
