# Supplementary figures and images for: Single-Cell Transcriptome Profiling Revealed That Vitrification of Somatic Cloned Porcine Blastocysts Causes Substantial Perturbations in Gene Expression
Source: Front Genet. 2020 Jul 24;11:640. doi: 10.3389/fgene.2020.00640 (PMC7394247; doi:10.3389/fgene.2020.00640)

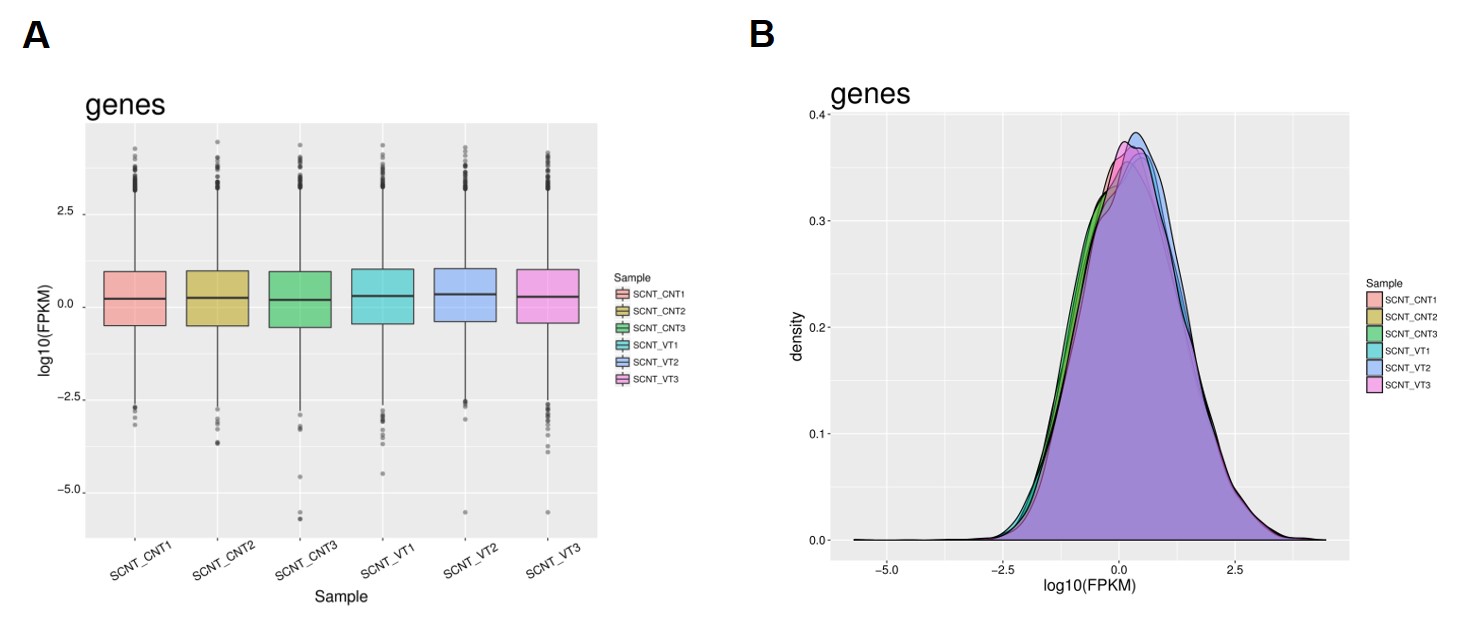

Supplement: Supplementary Figure 1 — RNA-Seq data quality. The boxplot (A) shows the gene expression level [log10(FPKM)] for each sample. (B) Density maps for different samples. [file Image_1.JPEG]

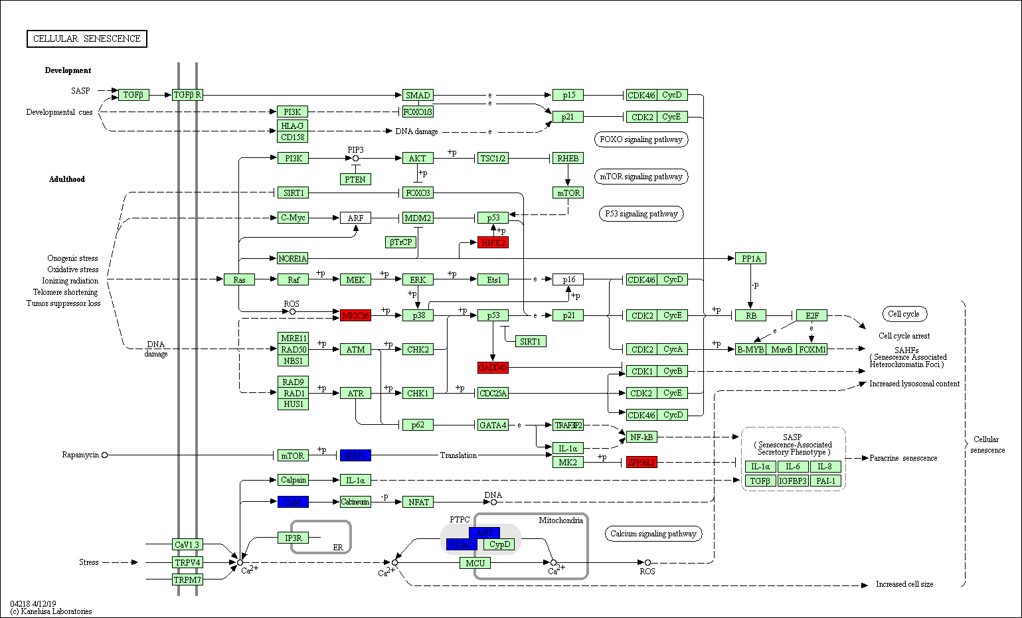

Supplement: Supplementary Figure 2 — Differentially expressed genes in vitrified/thawed porcine cloned blastocysts (SCNTVT) that were involved in the cellular senescence pathway. DAVID analysis showed that the cellular senescence pathway was significantly influenced (p = 0.037). Red indicates genes upregulated in the SCNTVT group: HIPK3, MAP2K3, GADD45B, and ZFP36L1. Blue indicates genes downregulated in the SCNTVT group: EIF4EBP1, CALML5, VDAC3, and SLC25A4. [file Image_2.JPEG]
